# Predictive factors in patients with advanced and metastatic squamous cell carcinoma of the head and neck: A study based on SWOG protocol S0420

**DOI:** 10.3892/or.2013.2374

**Published:** 2013-04-02

**Authors:** SMITA MEHTA, JAMES MOON, MEHMOOD HASHMI, MICHAEL LEBLANC, CHAO HUI HUANG, ELIZABETH RINEHART, GREGORY T. WOLF, SUSAN G. URBA, SUSHANTA K. BANERJEE, STEPHEN WILLIAMSON

**Affiliations:** 1 Department of Hematology and Oncology, University of Kansas Medical Center, Westwood, KS 66205, USA; 2 Cancer Research Unit, Kansas City VA Medical Center, Kansas City, MO 64128, USA; 3 SWOG Statistical Center, Seattle, WA 98109, USA; 4 Medical Center, University of Michigan, Ann Arbor, MI 48109, USA; 5 Department of Anatomy and Cell Biology, University of Kansas Medical Center, Kansas City, KS 66103, USA

**Keywords:** head and neck metastatic squamous cell carcinoma, sorafenib, NRP-1, HER-2

## Abstract

To evaluate the prognostic values of different protein expression in the progression of squamous cell carcinoma of the head and neck (SCCHN) patients, we conducted immunohistochemical (IHC) analysis in tissue samples of different patients enrolled on SWOG protocol S0420. S0420 was a phase II trial to evaluate the efficacy and safety of single-agent sorafenib in chemotherapy-naïve patients with metastatic or recurrent SCCHN. The primary end point was response probability, i.e., confirmed complete (CR) and partial response (PR). Sorafenib was administered orally at 400 mg twice daily on a continuous basis in 28-day cycles to eligible patients. Responses were evaluated according to RECIST (Response Evaluation Criteria in Solid Tumors) criteria. IHC analysis was performed for various markers and data were analyzed statistically. IHC data were obtained from 19 patients enrolled on S0420. There was a high frequency of cases with expression of the angiogenesis markers SMA, HIF-1α, Raf-1, VEGF and VEGF-R. None of the markers were significantly associated with response. Negative HER-2 status was associated with longer progression-free survival (PFS), P=0.04. Negative NRP-1 status was associated with longer overall survival (OS), P=0.04. There were no other significant associations. An almost universal overexpression of angiogenesis markers in the samples analyzed supports the evaluation of angiogenesis inhibition as a potential target for therapy. High levels of NRP-1 and HER-2 in SCCHN samples appear to be associated with decreased survival and earlier progression of disease, respectively, in SCCHN patients and may represent targets for therapy.

## Introduction

Squamous cell carcinoma of the head and neck (SCCHN) has a poor prognosis in recurrent and metastatic settings, with a median survival of approximately 6–8 months (1). Studies have demonstrated median progression-free survival (PFS) of 3 months and median overall survival (OS) of 7 months when single agent chemotherapeutic regimens were used (2–4). Current treatment strategies include single agent chemotherapy, combination chemotherapy and radiotherapy, targeted agents (either alone or in combination with conventional chemotherapy) and best supportive care (5,6). A SWOG phase III trial using cisplatin and 5-FU demonstrated superiority over single agents, in terms of response rates (21 vs. 10%) but at the expense of increased toxicity and with no survival benefit (7). A phase III, ECOG trial established similar responses for cisplatin and paclitaxel when compared to cisplatin and 5-FU (8). When the epidermal growth factor receptor (EGFR) inhibitor cetuximab was added to combination chemotherapy, response rates increased from 20 to 36% while PFS and OS improved to 5.6 and 10.1 months vs. 3.3 and 7.4 months, respectively (6). There is an immediate need to develop new agents with activity in this disease and to understand the mechanisms that interfere with the efficacy of current regimens. Knowledge of new pathways and factors that may predict for response to new agents can provide potential targets for future development of drugs with the potential for improved efficacy and reduced toxicity.

Sorafenib has demonstrated a broad range of antitumor activity in xenograft models (9). Sorafenib is an inhibitor of wild-type and mutant B-Raf and c-Raf kinase isoforms *in vitro*(10,11). Sorafenib also inhibits, *in vitro*, several receptor tyrosine kinases including vascular endothelial growth factor receptor (VEGFR)-2, murine VEGFR-2, murine VEGFR-3, murine platelet-derived growth factor receptor (PDGFR), Flt-3, c-KIT and p38 (MAPK family) (10,11). Elser *et al*(10) performed biomarker analysis on paired tumor samples from patients with previously treated head and neck cancer before and after treatment with sorafenib and found decrease in pERK, decrease in Ki67 and downregulation of Mcl-1 after treatment with sorafenib. Sorafenib has also received approval for treatment of advanced clear cell renal cancer, clinically demonstrating a benefit from inhibition of angiogenesis (12).

We have previously reported the results of a phase II trial evaluating the efficacy and safety of single agent sorafenib in chemotherapy naïve patients with metastatic or recurrent SCCHN (11). Fresh or archival tissue was requested at the time of enrollment on S0420 and biopsies of tumor tissue at the time of progression were requested for subsequent biomarker analysis. We intended to evaluate different biomarkers of angiogenesis pathways, EGFR and Ras-Raf pathway by IHC. This report summarizes the findings from the biomarker analysis.

## Materials and methods

### Patient samples and treatment profile

In the phase II SWOG S0420 study, we enrolled chemotherapy naïve patients with metastatic, persistent or recurrent squamous cell carcinoma of head and neck (SCCHN) (11).

These patients received sorafenib orally at 400 mg twice daily on a continuous basis, in 28-day cycles. Responses were evaluated according to RECIST criteria. Fresh or archival tissue was requested at the time of enrollment on S0420 and biopsies of tumor tissue at the time of progression were requested for subsequent paired specimen biomarker analysis, to evaluate for treatment effects on the tumor. We performed retrospective IHC analysis on the patient’s cancer tissue samples for multiple markers. These included neuropilin-1 (NRP-1), HER-2/neu, VEGF, VEGF receptor, Raf-1, epidermal growth factor receptor (EGFR), smooth muscle actin (SMA), hypoxia inducible factor-1α (HIF-1α), E-cadherin, P-27 and cyclin D1. One of the limitations for this study was lack of post-treatment tumor samples from almost all patients. We received baseline specimens from 25 patients, but obtained paired specimens, before and after treatment with sorafenib, from only one of those patients. However, specimens received from only 19 patients were of sufficient quality and quantity for adequate analysis of any biomarker. All patients gave written informed consent. Approval from the institutional ethics review boards of all participating centers were obtained before the study.

### Reagents

Anti-VEGF, anti-VEGFR antibodies were purchased from Thermo Fisher Scientific (USA). Anti-HIF-1α, anti-NRP-1, anti-P-27 and anti-HER-2/neu were obtained from Santa Cruz Biotechnology, Inc. (CA, USA). Anti-Raf-1 and anti-SMA were obtained from Abcam (MA, USA). Anti-E-cadherin and anti-EGFR were obtained from BD Transduction (MD, USA). Anti-cyclin D1 was obtained from Oncogene Research Products (CA, USA). IHC kit was obtained from Zymed Laboratories (CA, USA). Except where otherwise specified, all reagents were obtained from Sigma Chemical Co. (USA).

### Immunohistochemistry

Immunohistochemical analysis was performed according to our previous method (13,14). The tissue sections were collected, then shipped from the various participating SWOG sites to the Kansas City Veterans Affairs Medical Center (KCVAMC) and stored at -40°C. Paraffin-embedded tissue blocks were cut in the Department of Pathology as per the guidelines followed by KCVAMC. The immunohistochemistry was performed on 4% formalin-fixed, paraffin-embedded tissue sections. Briefly, tissue sections were de-paraffinized in xylene, rehydrated in different grades of alcohol from 100% to 50%, then washed with phosphate-buffered saline (PBS) and blocked with tissue blocker (Zymed Laboratories, CA) for 10 min and immunostained by specific antibodies with overnight incubation. The tumor sections obtained from database were reviewed and reconfirmed by a pathologist using adjacent hematoxylin and eosin stained slides.

### Statistical analysis

Response rates between positive and negative groups were compared using two-sided Fisher’s exact test. PFS and OS were estimated using the method of Kaplan-Meier, and were compared between groups using a two-sided log-rank test. A P-value of ≤0.05 was considered significant. Because this was an exploratory analysis, no adjustment of P-values was made for multiple comparisons. Baseline patient characteristics were compared between patients registered to S0420 for whom we did not receive usable tissue and patients included in this analysis. Race and gender were compared using a two-sided Fisher’s exact test. Age was compared using a two-sided t-test.

## Results

### Forty-four patients were enrolled in SWOG S0420

**Figure 1:**
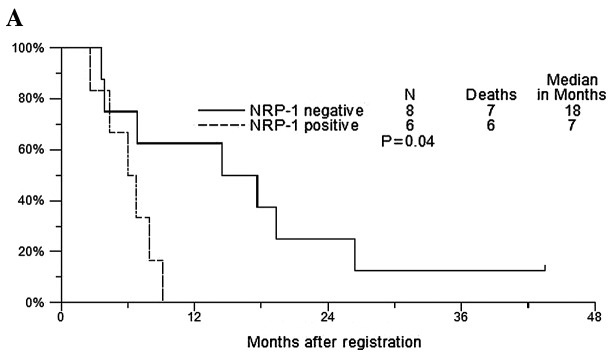

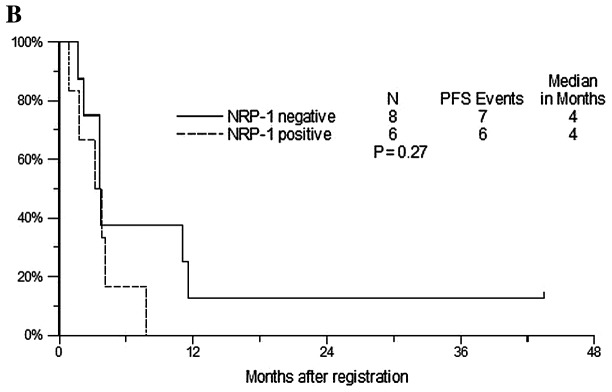
(A) Overall survival by NRP-1 status. (B) Progression-free survival by NRP-1 status.

**Figure 2:**
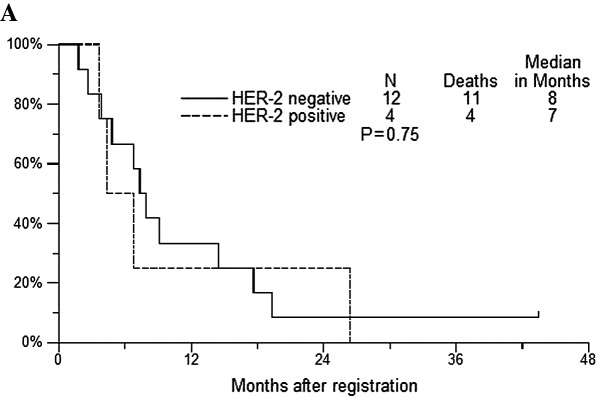

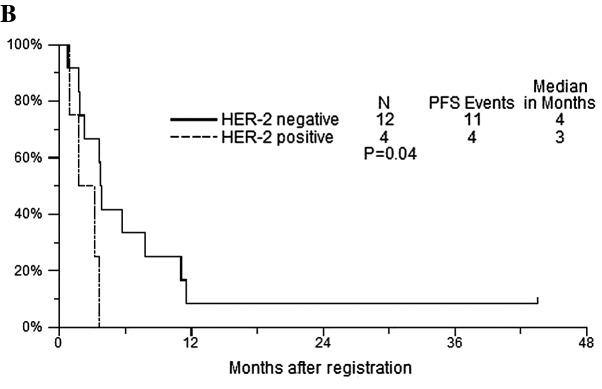
(A) Overall survival by HER-2 status. (B) Progression-free survival by HER-2 status.

Forty-two eligible patients were assessed for efficacy and adverse events. Not all of the tissues collected were of sufficient quality and quantity, thus IHC data were available on only 19 patients (Table I). There is discrepancy in the number of samples of IHC for each biomarker depending on the availability and quality of the sample. IHC for NRP-1 and HER-2 were performed on 14 samples and Raf-1 was performed in 11 samples due to the limited amount of quality tissue. Results are shown in Table I and II. Expression of the angiogenesis markers SMA, Raf-1, HIF-1α, VEGF and VEGF-R were positive in 100, 100, 93, 83 and 69% of specimens evaluated, respectively. Expression of NRP-1 was seen in 43% (6/14) of patients. None of the markers were significantly associated with response. Negative NRP-1 status was associated with longer OS of 18 vs. 7 months (P=0.04) (Fig. 1). Expression of HER-2 was seen in 25% (4/16). Negative HER-2 status was associated with longer PFS of 4 vs. 3 months (P=0.04) (Fig. 2). For SMA and HIF-1α markers, the negative groups were too small to make any statistically significant comparisons. There were no other significant associations at the nominal 0.05 level.

## Discussion

This study was conducted to evaluate the role of sorafenib in the treatment of recurrent and/or metastatic SCCHN. There was strong rational for this approach since sorafenib targets EGFR-Ras-Raf-MEK-ERK signaling pathway as well as angiogenesis. We have previously reported the results of S0420 which demonstrated that sorafenib is reasonably well tolerated in patients with advanced or metastatic SCCHN (11). Our trial and another trial by Elser *et al*(10) demonstrated a modest level of activity of sorafenib as a single agent in SCCHN. The Elser trial performed biomarker analysis on paired tissues before and after treatment with sorafenib and demonstrated, in a preliminary fashion, a biologic effect of the drug with evidence of disruption of the EGFR-Ras-Raf-MEK-ERK signaling pathway, a pro-apoptotic effect, and possibly, an effect on angiogenesis pathways, which were also overexpressed in all 5 of their samples (10).

Development of this agent in SCCHN will be dependent on additional correlative studies to define potential pretreatment biologic markers that may predict for response to sorafenib. In this study we evaluated the angiogenesis biomarkers VEGF, NRP-1, VEGF receptor and HIF-1α and SMA. One important finding of our biomarker analysis is that we found almost universal expression of these markers of angiogenesis in the samples we analyzed and thus provides further support for the evaluation of agents that disrupt angiogenesis as a potential target for treating advanced SCCHN.

Vascular endothelial growth factor and receptors are important regulators of vasculogenesis, angiogenesis, lymph angiogenesis and vascular permeability (15,16). VEGF and other cell surface receptors play a major role in the development of metastasis and poor survival associated with various cancers including SCCHN cancers (17). Out of multiple VEGF isomers, two forms VEGF_121_ and VEGF_165_ are the most common. VEGF_165_ interacts with non-signaling Neuropilin co-receptors (18,19). VEGF_165_ has been identified as an inducer of pathological neo-vascularization (18–22). Neuropilin-1 (NRP-1) is a cell-surface receptor for VEGF_165_ and class 3 semaphorins is expressed by neurons and endothelial cells and acts as a mediator of angiogenesis and neuronal guidance (18,19). It is overexpressed by many cancers and is associated with increased neo-angiogenesis and aggressive tumor behavior (23–26). Neuropilin-1 is expressed in various tumor cells such as breast, prostate, lung, melanoma cells and acute myeloid leukemia (27–30). This tumor cell derived NRP-1 is functionally active and may act as a positive modulator of tumor angiogenesis and a negative regulator of tumor cell apoptosis in the presence or absence of VEGF. It has also been reported that NRP-1 is an independent predictor of cancer relapse and poor survival in patients with non-small cell lung cancer, similar to our finding in our population of SCCHN (31). A computational model predicts *in vivo* efficacy of several neuropilin-targeted compounds. This model predicts that blockade of neuropilin-VEGFR coupling is significantly more effective than other approaches in decreasing VEGF-VEGFR-2 signaling (32,33). NRP-1 was studied in laryngeal carcinoma and found to be expressed in laryngeal squamous cell carcinoma tissues by IHC and all laryngeal cell lines by RT-PCR (34).

It is interesting that in our study there was no differential effect associated with expression of VEGF, however, a negative effect was noted with overexpression of NRP-1. This would suggest that overexpression of NRP-1 may predict for resistance to the inhibition of angiogenesis by sorafenib and that angiogenesis inhibitors with a different mechanism of action are needed in patients whose tumors overexpress NRP-1. Further studies using NRP-1 receptor inhibitors concurrently with sorafenib may be warranted in an attempt to increase the sensitivity of sorafenib. Further basic research with NRP-1 and identification of the exact mechanism for NRP-1inhibitors as inhibitors of angiogenesis are needed (29,30). Another approach would be to evaluate the activity of sorafenib with other agents in patients whose tumors do not overexpress NRP-1. These findings need to be further explored through basic research conducting *in vitro* studies and using xenograft models.

Epidermal growth factor receptor (EGFR) is overexpressed in several epithelial malignancies, including SCCHN, which exhibits EGFR overexpression in up to 90% of tumors (35–37). Inhibition of EGFR by the monoclonal antibody, cetuximab has been shown to improve PFS and OS when combined with chemotherapy in patients with advanced head and neck cancer (6,36,37).

Our study demonstrated that the negative HER-2 status was associated with longer PFS, P=0.04. The prognostic significance of overexpression of HER-2 in head and neck cancer patients has been evaluated with conflicting results, however, most studies suggest a negative impact on freedom from disease and survival, similar to our findings (38). The combination of EGFR, HER-2/neu and HER-3 expression is a stronger predictor for the outcome of oral squamous cell carcinoma than any individual isoform (39,40). Perhaps by blocking EGFR and/or HER-2 signaling pathway, we can increase the sensitivity of head and neck cancer cells to sorafenib. We would propose further basic and clinical research exploring combinations EGFR and HER-2 inhibitors in SCCHN tumors overexpressing HER-2.

One major limitation of our study was the inconsistent quality and quantity of the samples. Due to lack of post-treatment samples it is very difficult to make concrete conclusions. Further biomarker studies on a larger number of patients are warranted to confirm our findings and correlate them with clinical outcome.

We conclude that our finding of nearly uniform overexpression of markers associated with angiogenesis provides further support for exploring angiogenesis inhibitors in SCCHN. In addition, NRP-1 and HER-2/neu expression have a negative predictive value for overall survival and progression-free survival, respectively, in this population of squamous cell carcinoma of head and neck treated with sorafenib and may predict resistance to this agent, however, further studies are needed.

## Figures and Tables

**Table I:** Frequencies and responses for the IHC results.

	IHC results
	
	Frequency (%)
VEGF	
Negative	3 (17)
Positive	15 (83)
HER-2	
Negative	12 (75)
Positive	4 (25)
NRP-1	
Negative	8 (57)
Positive	6 (43)
E-cadherin	
Negative	3 (23)
Positive	10 (77)
P-27	
Negative	5 (31)
Positive	11 (69)
VEGFR-1	
Negative	5 (31)
Positive	11 (69)
EGFR	
Negative	10 (63)
Positive	6 (38)
Cyclin D1	
Negative	4 (27)
Positive	11 (73)
HIF-1α	
Negative	1 (7)
Positive	13 (93)
SMA	
Negative	0 (0)
Positive	19 (100)
Raf-1	
Negative	0 (0)
Positive	11 (100)

**Table II:** Comparison of baseline patient characteristics between patients included in IHC analysis vs. those with no tissue available.

	No tissue	Provided tissue	P-value
Gender, n (%)			0.21
Male	21 (91)	14 (74)	
Female	2 (9)	5 (26)	
Race, n (%)			0.20
White	23 (100)	17 (89)	
Black	0 (0)	2 (11)	
Performance status^a^, n (%)			0.20
0	11 (50)	5 (26)	
1	11 (50)	14 (74)	
Age			0.03
No.	23	19	
Mean	60.0	67.8	
SD	10.8	11.2	
Median	61.1	66.9	
Minimum	31.1	48.8	
Maximum	82.2	84.2	

a Performance status was not reported for one patient.

SD, standard deviation.
